# Effect of the 5-HT_2C_ Receptor Agonist WAY-163909 on Serotonin and Dopamine Metabolism across the Rat Brain: A Quantitative and Qualitative Neurochemical Study

**DOI:** 10.3390/ijms20122925

**Published:** 2019-06-14

**Authors:** Sara Whitestone, Philippe De Deurwaerdère, Lynn Baassiri, Julien Manem, Youssef Anouar, Giuseppe Di Giovanni, Rahul Bharatiya, Abdeslam Chagraoui

**Affiliations:** 1 Centre National de la Recherche Scientifique (Unité Mixte de Recherche 5287), 146 rue Léo Saignat, B.P.281, F-33000 Bordeaux CEDEX, France; sarawhitestone@gmail.com (S.W.); deurwaer@u-bordeaux.fr (P.D.D.); lynnbaassiri@gmail.com (L.B.); julien.manem@gmail.com (J.M.); bhartiyarahul20@gmail.com (R.B.); 2 Neuronal and Neuroendocrine Differentiation and Communication Laboratory, Institute for Research and Innovation in Biomedicine of Normandy (IRIB), Normandie Univ, UNIROUEN, INSERM, U1239, CHU Rouen, 76000 Rouen, France; youssef.anouar@univ-rouen.fr; 3 Department of Medical Biochemistry, Rouen University Hospital, 76000 Rouen, France; 4 Department of Physiology and Biochemistry, Faculty of Medicine and Surgery, University of Malta, MSD 2080 Msida, Malta; giuseppe.digiovanni@um.edu.mt; 5 Neuroscience Division, School of Biosciences, Cardiff University, Cardiff CF10 3AT, UK; 6 Department of Biomedical Sciences, Section of Neuroscience and Clinical Pharmacology, University of Cagliari, SS 554, km 4,500, 09042 Monserrato, Cagliari, Italy

**Keywords:** metabolism, dopamine, serotonin, 5-HIAA, HPLC, HVA, DOPAC, correlative analysis, 5-HT_2C_ receptor, connectivity

## Abstract

The effects triggered by serotonin2C (5-hydroxytryptamin_2C_, 5-HT_2C_) receptor agonists in the brain are often subtle, and methodologies highlighting their widespread actions to account for their multiple modulatory influences on behaviors are still lacking. We report an extended analysis of a neurochemical database on monoamines obtained after the intraperitoneal administration of the preferential 5-HT_2C_ receptor agonist WAY-163909 (0.3 and 3 mg/kg) in 29 distinct rat brain regions. We focused on the metabolite of 5-HT, 5-hydroxyindoleacetic acid (5-HIAA), the metabolites of dopamine (DA), 3,4-dihydroxyphenylacetic acid (DOPAC) and homovanillic acid (HVA), and the index of the turnovers 5-HIAA/5-HT and DOPAC/DA. WAY-163909 increased and decreased 5-HIAA tissue levels in the amygdala and dorsolateral orbitofrontal cortex, respectively, and decreased the 5-HT turnover in the infralimbic cortex. It enhanced HVA levels in the medial orbitofrontal cortex and DOPAC levels in the amygdala. WAY-163909 increased and decreased DA turnover in the medial orbitofrontal cortex and the anterior insular cortex, respectively. The correlative analysis of the turnovers between pairs of brain regions revealed low levels of correlations across the brain but presented a distinct pattern of correlations after WAY-163909 was compared to saline-treated rats. WAY-163909, notably at 0.3 mg/kg, favored cortico-cortical and cortico-subcortical correlations of both turnovers separately, and frontal DOPAC/DA ratio with cortical and subcortical 5-HIAA/5-HT ratios at 3 mg/kg. In conclusion, the qualitative, but not the quantitative analysis shows that WAY-163909 alters the pattern of correlations across the brain, which could account for its multiple behavioral influences.

## 1. Introduction

The serotonin2C (5-hydroxytryptamin_2C_, 5-HT_2C_) receptor, a seven-transmembrane receptor coupled to several intracellular signaling pathways [1], is thought to act as a “neural rheostat regulating the intersection between vulnerability behaviors” [2]. This hypothesis parallels the initial proposal that 5-HT_2C_ receptors regulate or even inhibit the excitability of neurobiological networks [3]. The ability of 5-HT_2C_ receptors to shape neurobiological networks is widespread, and the receptor has been shown to interfere with both the serotonin (5-HT) and dopamine (DA) systems in the mammalian brain. The stimulation of 5-HT_2C_ receptors has been shown in rodents to reduce feeding behavior [4,5], drug intake [6,7,8], impulsive behavior [9,10], or psychosis [11] and to trigger anxiety [12,13,14] and compulsive forms of behaviors [15,16], to cite a few [1]. It could also participate in the mechanism of action of some hallucinogenic drugs, including d-Lysergic acid diethylamide [17]. The understanding of 5-HT_2C_ receptor function is critical for the development of therapeutic drugs for the treatment of depression, schizophrenia, and drug abuse [1,18], but it presents a major neurobiological challenge.

The widespread expression of the 5-HT_2C_ receptor subtype in the CNS [19,20,21,22], and the paucity of the responses to 5-HT_2C_ receptor agonists obtained with classical neuroanatomical markers of neuronal activity, such as c-Fos [23,24,25], makes it difficult to study the consequences of its stimulation on neurobiological networks. There are numerous data reporting that the stimulation of 5-HT_2C_ receptors alters the activity of neuronal cells in brain tissues *in vitro* and *in vivo* [1,23]. However, it is also difficult to understand the relationship between these neuronal responses to 5-HT_2C_ receptor stimulation, their consequences on a neurobiological network and, ultimately, the behaviors. The ascending 5-HT systems of the dorsal and median raphe nuclei (DR and MR) innervate the brain quite diffusely, while the ascending DA systems of the substantia nigra (SN) and ventral tegmental area (VTA) have a smaller number of prescribed circuits [26]. The biochemical activity at their terminal fields includes both homologous and heterologous controls, the latter involving neighboring cells interacting with neurobiological networks [27,28]. The neurochemical approach of analyzing tissue monoamine content across several parts of the brain is a possibility to study extrinsic influences operating on terminals of monoaminergic neurons.

Recently, we reported that the preferential agonist WAY-163909 modified the tissue content of 5-HT and DA only in a few brain regions. However, it reduced the correlations of DA tissue content established between pairs of brain regions and redistributed the pattern of correlations for DA and 5-HT content across the brain [29]. The measurement of metabolites of monoamines can inform other aspects of monoamine neuron function compared to the neurotransmitter itself [30]. Previous studies have shown clear regional differences in basal neurotransmitter release, metabolite levels, and metabolite turnover rates [31,32,33]. 5-hydroxyindoleacetic acid (5-HIAA) and 3,4-dihydroxyphenylacetic acid (DOPAC) come from the breakdown of 5-HT and DA, respectively, via a first oxidation by monoamine oxidase A (MAO-A) followed by aldehyde dehydrogenase [34]. Homovanillic acid (HVA) is the final product of the degradation of DA involving the catechol-O-methyl transferase (COMT) [27,34]. In contrast to the neurotransmitter tissue content, mainly corresponding to compounds contained in vesicles of exocytosis at terminals [27], metabolites also come from the activity of surrounding cells. DOPAC can be produced both inside and outside catecholaminergic cells. For 5-HIAA, MAO-A is not often present at terminals of 5-HT neurons; therefore, this process likely occurs outside the terminals in the case of 5-HT [27,35,36,37]. Similarly, COMT is located elsewhere other than catecholaminergic cells [38]. Meanwhile, the 5-HIAA/5-HT and DOPAC/DA ratios, which are considered as indirect indexes of the turnover, vary across the brain, and the pattern of correlations between pairs of specific brain regions is different for the parent neurotransmitter [32,39]. It is, therefore, possible that the stimulation of central 5-HT_2C_ receptors alters the metabolism of 5-HT and DA in a different way compared to the neurotransmitter itself.

In the present study, we have expanded the analysis of the effect of the selective 5-HT_2C_ receptor agonist WAY-163909 (0.3 and 3 mg/kg, intraperitoneally (i.p.)) on 5-HT and DA function by studying its quantitative and qualitative neurochemical impact on metabolites of 5-HT and DA, as well as their turnovers. The analysis was done across 29 brain regions, including the cortex, basal ganglia, mesencephalon, amygdala, hippocampus, and hypothalamus.

## 2. Results

### 2.1. Quantitative Analysis of DA and 5-HT Metabolism after WAY-163909 Treatment

**Figure 1:**
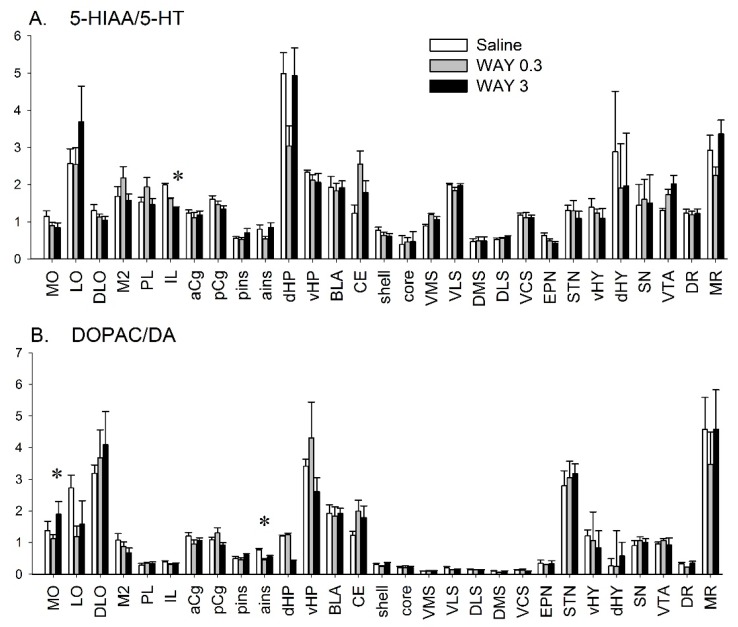
Effect of WAY-163909 on turnover of 5-HT (5-HIAA/5-HT ratio) (**A**) and DA (DOPAC/DA ratio) (**B**) across rat brain regions. The left, medial, and right panels correspond to saline-, WAY-163909 0.3 mg/kg, and 3 mg/kg treated rats, respectively. The results correspond to the mean ± SEM of the ratios in 29 different brain regions. WAY-163909 or saline has been intraperitoneally (i.p.) administered, and the tissue values correspond to 45 minutes after the administration. Data for DA and 5-HT tissue contents used in this experiment have been already reported [29]. WAY-163909’s effects have been compared to saline-treated rats using a one-way ANOVA. The number of values considered has been reported in Table S1. * *p* < 0.05 with respect to the saline treatment group (protected least significant difference (PLSD) test).

We studied the effect of WAY-163909 treatment (0.3 and 3 mg/kg i.p.) on DA and 5-HT metabolism in 29 brain areas of rats and compared it with saline-treated rats (8 rats in each of the three groups; n = 24 for the entire experiment) (Figure 1). This postmortem analysis corresponds to 45 minutes after the injection of WAY-163909 (see Methods). The quantitative distribution of tissue 5-HIAA paralleled that previously reported for 5-HT [29]. The highest 5-HIAA level was found in the SN (738 ± 55.9 pg/mg) (Table 1). The ratio of 5-HIAA/5-HT was also heterogeneous in brain ranging from low (dorsomedial striatum (DMS), dorsolateral striatum (DLS), pins, ains) to high in the MR, dorsal hippocampus (dHP), and lateral orbitofrontal cortex (LO) (Figure 1A).

WAY-163909 (0.3 mg/kg) reduced 5-HIAA levels in the dorsolateral orbitofrontal cortex (DLO) and increased them in the central nucleus of the amygdala (CE) (F(2,23) = 3.61, *p* < 0.05, and F(2,22) = 5.307, *p* < 0.05, respectively) (Table 1). WAY-163909 decreased the ratio of 5-HIAA/5-HT in the infralimbic cortex (IL) (F(2,23) = 3.694, *p* < 0.05) at 3 mg/kg only (Figure 1A).

The pattern of quantitative distribution of the two main metabolites of DA, namely, DOPAC and HVA, is similar to the parent neurotransmitter [29], very concentrated in the striatum and NAc, substantially concentrated in the SN or VTA, and poorly present in other brain regions including the various cortical, hippocampal, and hypothalamic subregions (Table 1). The levels of HVA were also below the threshold of detection in several brain regions, in part due to the lower electrochemical sensitivity of our apparatus for HVA compared to DOPAC or DA (see methods). The DOPAC/DA ratio was heterogeneous between the structures of the brain. It was low in the striatum (close to 0.1 and 0.15 or 0.2 in the nucleus accumbens) and high in some cortical areas, the subthalamic nucleus (STN), the hippocampus or the MR (Figure 1B).

WAY-163909 did not alter DOPAC levels in any regions whereas it increased HVA tissue content in MO (F(2,23) = 3.61, *p* < 0.05) only (Table 1). The effect was observed for both doses of WAY-163909 with a similar magnitude. WAY-163909 modified the DA turnover in the MO (F(2, 22) = 3.53, *p* < 0.05) and ains (F(2,18) = 12.2, *p* < 0.001). Specifically, the DA turnover was increased in MO at a dose of 3 mg/kg and decreased in ains at a dose of 0.3 mg/kg (Figure 1B).

#### 2.1.1. Pattern of Correlations of the 5-HIAA/5-HT Ratio across the Brain

**Figure 2:**
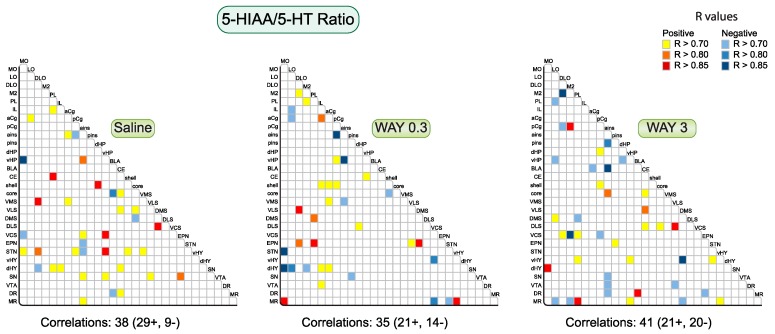
Correlative analysis of neurochemical indexes of 5-HT turnover (5-HIAA/5-HT ratio) across rat brain regions. Representation of the range of Pearson’s *r* values for each linear regression, 5-HIAA/5-HT ratio between the 29 brain areas in saline, WAY-163909 0.3 mg/kg, and 3 mg/kg treated rats. Colored boxes correspond to the existence of a significant correlation between the two parameters (yellow to red: positive; blue: negative) considered after correction for multiple comparisons.

**Figure 3:**
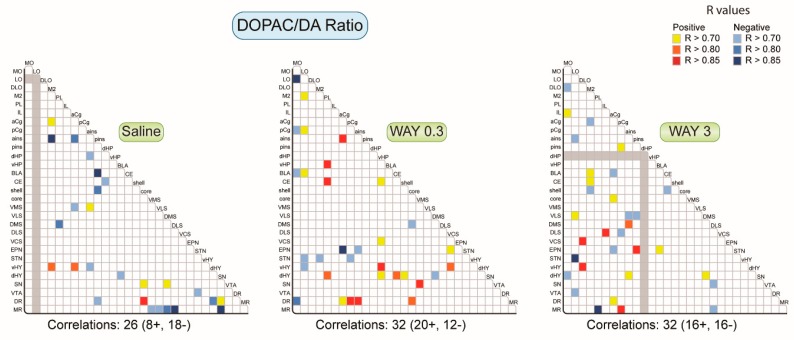
Correlative analysis of neurochemical indexes of DA turnover (DOPAC/DA ratio) across rat brain regions. Representation of the range of Pearson’s *r* values for each linear regression, DOPAC/DA ratio between the 29 brain areas in saline, WAY-163909 0.3 mg/kg, and 3 mg/kg treated rats. Colored boxes correspond to the existence of a significant correlation between the two parameters (yellow to red: positive; blue: negative) considered after correction for multiple comparisons. The grey boxes indicate that these correlations were not performed due to the low number of values (see Table S1).

We evaluated the effect of WAY-163909 on 5-HT metabolism by studying the correlations of the 5-HIAA/5-HT ratio between pairs of regions across the 29 sampled brain regions (Figure 2). The correlations have been corrected as previously reported (see Methods). In saline-treated rats, we reported 38 significant correlations for the 5-HIAA/5-HT ratio equilibrated between positive (29) and negative (9) correlations. This represents about 9% of pairs of the entire analysis. Regions such as STN or ains established several correlations with other brain regions. Interestingly, the number of correlations remained almost constant after WAY-163909 (35 comprising 21 positive and 14 negative correlations after 0.3 mg/kg; 41 comprising 21 positive and 20 negative correlations after 3 mg/kg), but the pattern of correlations was different among the treatment groups. WAY-163909 enhanced the correlations for the 5-HT turnover in IL, aCg, shell, and entodepuncular nucleus (EPN) at 0.3 mg/kg compared to saline-treated rats. By contrast, it enhanced the correlations in LO, M2, and MR at 3 mg/kg. These changes of pattern induced by WAY-163909 occurred at the expense of the correlations in the STN, as well as the CE and SN. WAY-163909 also changed the pattern of correlations found in the dHY at 0.3 mg/kg compared to saline-treated rats, with correlations centered no longer on the dHY but the ventral hypothalamus (vHY) instead at 3 mg/kg. To summarize, from a heterogeneous distribution in saline-treated rats, WAY-163909 favored correlations between cortical and subcortical areas at 0.3 mg/kg, but not at 3 mg/kg (Figure 3).

#### 2.1.2. Pattern of Correlations of the DOPAC/DA Ratio across the Brain

We evaluated the effect of WAY-163909 on the DOPAC/DA ratio. The saline-treated rats showed 26 correlations for the DOPAC/DA ratio comprising 8 positive and 18 negative correlations (Figure 3). The distribution did not reveal a specific pattern or organization, except the slightly higher number of correlations established with the MR, and did not show the expected correlations between the quadrants of the striatum. WAY-163909 slightly increased the number of correlations to 32 at both 0.3 and 3 mg/kg. Beyond the balance of positive/negative correlations, which was different between the two doses (20 positive and 12 negative correlations at 0.3 mg/kg versus 16 positive and 16 negative correlations at 3 mg/kg), the pattern of correlations also differed. WAY-163909 (0.3 mg/kg) suppressed the number of correlations as compared to saline-treated rats in the M2, SN, and MR. Conversely, WAY-163909 potentiated the correlations in the orbitofrontal cortex (MO, LO), PL, vHP, EPN, and STN, which was completely absent in saline-treated rats. It is also noticeable that WAY-163909 (3 mg/kg) increased the correlation between the striatal DOPAC/DA ratio with that of the frontal cortex, which was not seen in saline and WAY-163909 (0.3 mg/kg)-treated rats. Thus, WAY-163909 favored correlations of DOPAC/DA from diencephalic/mesencephalic regions toward other brain regions at 0.3 mg/kg and from cortical regions toward cortical and subcortical regions at 3 mg/kg.

#### 2.1.3. Pattern of Correlations between DOPAC/DA and 5-HIAA/5-HT Ratio across the Brain

**Figure 4:**
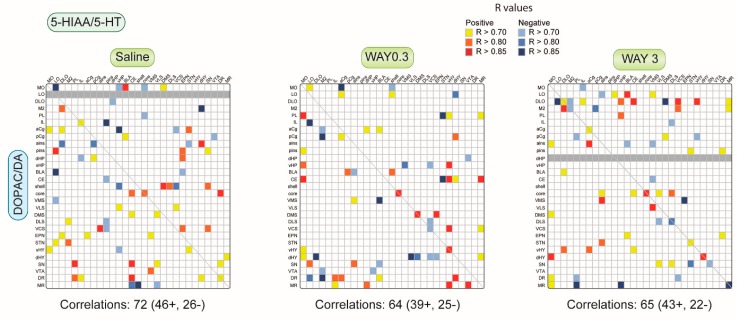
Correlative analysis between the 5-HT turnover (5-HIAA/5-HT ratio) and DA turnover (DOPAC/DA ratio) across rat brain regions. Representation of the range of Pearson’s *r* values for each linear regression of 5-HIAA/5-HT ratio (top horizontal) versus DOPAC/DA ratio (left vertical) within and between the 29 brain areas in saline (first column), WAY-163909 0.3 mg/kg (second column) and 3 mg/kg (third column) treated rats. Colored boxes correspond to the existence of a significant correlation between the two parameters (yellow to red: positive; blue: negative) considered after correction for multiple comparisons. The grey boxes indicate that these correlations were not performed due to the low number of values (see Table S1).

We looked at the correlations established between DA metabolism (DOPAC/DA ratio) and 5-HT metabolism (5-HIAA/5-HT ratio) across selected brain regions (Figure 4). The DOPAC/DA and 5-HIAA/5-HT ratios significantly correlated in 73 brain areas (comprising 47 positive and 26 negative correlations), representing again less than 10% of all analyses. It included 3 correlations in the same brain area (all positive) of saline-treated rats. The correlations included the NAc core and shell, EPN, and SN. Interestingly, the number of correlations decreased after 0.3 mg/kg and 3 mg/kg WAY-163909 injection (64 and 65 respectively, mostly positive correlations). The proportion of correlations in the same brain region was significantly more in 0.3 mg/kg WAY-163909 treated rats 7 (5 positive) compared to saline-treated rats. WAY-163909 completely diminished the correlation of the NAc shell DOPAC/DA ratio with the 5-HIAA/5-HT ratio in the striatum and SN. Likewise, WAY-163909 also suppressed the correlation between the DOPAC/DA ratio with the 5-HIAA/5-HT ratio of frontal cortex in basal ganglia mesencephalon (EPN and STN), as well as the basal ganglia mesencephalon (EPN and STN) DOPAC/DA ratio with the orbitofrontal cortex (LO, DLO, and M2). The number of correlations dramatically increased in the CE and hypothalamus after WAY-163909 (0.3 mg/kg) treatment. WAY-163909 (3 mg/kg) potentiated the correlation of DOPAC/DA ratio from DLO and M2 with different brain areas such as the cortex, hippocampus, amygdala, and striatum (DMS, VMS, VCS), which are absent in saline and WAY-163909 (0.3 mg/kg) treated rats. WAY-163909 (0.3 and 3 mg/kg) also considerably potentiated the correlation of DA turnover with 5-HT turnover in MR, which is absent in saline-treated rats. Markedly, WAY-163909 at 0.3 mg/kg enhanced the correlations involving both the dHY and the vHY.

## 3. Discussion

The results show that the 5-HT_2C_ receptor agonist WAY-163909 modifies the metabolism of 5-HT and DA systems in very few brain regions, with a noticeable increase in HVA content in the medial orbitofrontal cortex. Beyond quantitative, neurochemical changes, the results obtained with the correlations suggest that WAY-163909 rebalances the pattern of correlations of tissue monoamine metabolism in the brain.

The study of the metabolites and turnovers of 5-HT and DA systems brings up additional information on the impact of WAY-163909 on these systems, notably the increase in HVA or the ratio of DOPAC/DA in the MO cortex. Even if the purpose of our approach is not to speculate on the meaning of these results in terms of extracellular levels of DA, an increase in HVA in cortical regions is often interpreted as a consequence of an increase in DA release. Indeed, the clearance of extracellular DA is poorly achieved by DA reuptake sites, expressed at low levels in the orbitofrontal cortex. This strengthens the role of cortical, ectopic COMT in the degradation of DA [40]. The increase in HVA content parallels the increase in the ratio of DOPAC/DA in a situation where DA tissue content was not significantly altered [29]. It is important to stress that these effects are specific in this region. Moreover, we confirmed with the previous data that the quantitative, neurochemical changes for DA function are rather modest: increased DA in the CE at 0.3 mg/kg and decreased DA in the dHY [29], in addition to the decrease in DOPAC/DA turnover in ains at 0.3 mg/kg.

The changes are also modest regarding the metabolism of the 5-HT system, since we reported opposite changes of 5-HIAA tissue content in DLO and CE after 0.3 mg/kg and a decrease in the 5-HT turnover only in the IL. The level of 5-HT was previously shown to be reduced in DLO at 0.3 mg/kg and increased in MO and M2 at 3 mg/kg. Previous data reported no modification on tissue content of 5-HT and turnover in a restricted number of regions with the 5-HT_2B/2C_ receptor agonist Ro-60-0175 [41].

It can be argued that tissue measurement of monoamine metabolism is less sensitive to detecting changes of monoamine function compared to more dynamic *in vivo* methods. Indeed, it has been shown on many occasions that the stimulation of central 5-HT_2C_ receptors inhibited the electrical activity of the VTA DA neurons [42,43] and DA release in the nucleus accumbens and/or the striatum [44,45,46], while likely not in the medial prefrontal cortex [47,48]. Nonetheless, it is now acknowledged that several other sources of 5-HT_2C_ receptors can modulate DA release, sometimes in an opposite manner [23,49,50], which may explain the differences in DA output according to the agonists used [1]. WAY-163909, probably one of the most selective 5-HT_2C_ receptor agonists available [1,51,52], does not modulate DA release in the nucleus accumbens or striatum at 1 mg/kg nor modify c-Fos expression in the basal ganglia except in the ventrolateral striatum (VLS) at 3mg/kg [24]. The effects of WAY-163909 on DA release occurred at higher doses increased in the cortex and decreased in nucleus accumbens [48]. Thus, even if the approach is less sensitive, it allows the study of monoaminergic metabolism in multiple regions encompassing several neurobiological networks. The correlation changes for the 5-HIAA/5-HT and DOPAC/DA ratios support the idea that WAY-163909 should trigger widespread effects in the brain while being behaviorally efficacious with the regimen we used for purposeless oral movements, body temperature, probabilistic reversal learning in mice, and food intake, to cite a few [24,48,53,54,55].

The existing correlations of the tissue content for a monoamine or its metabolism between two separated brain regions are not easily interpreted. In fact, the regulation of biochemical activity at terminals includes autoregulations and heteroregulations at the vicinity, the latter ones being included into neurobiological networks. A single DA neuron from the SN crosses many distinct networks and establishes thousands of presynaptic interactions with other neurons [56]. Speculatively, it is most likely that the biochemical activity in one part of the monoaminergic neuron does not correlate with another part of the same neuron. The tissue measurement of monoamines and metabolites grossly integrates all homologous and heterologous regulating mechanisms within the piece of tissue. It is not surprising that the level of correlations is low at least in rats that underwent behavioral tests without receiving any injection [32,39]. The changes of correlations of a parameter between two brain regions would indicate a change of allegiance of the terminals toward regulatory mechanisms impacting the activity of the terminals, either homologous or heterologous or both, even in the absence of net changes of quantities. WAY-163909 favored cortico-cortical and cortico-subcortical correlations for DOPAC/DA at both doses and for 5-HIAA/5-HT at 0.3 mg/kg. The correlations are very different between saline, 0.3, and 3 mg/kg WAY-163909 treated rats and do not go over 10% of the total analyses. A completely different pattern was also triggered by WAY-163909 for the parent neurotransmitter, although the number of correlations was reduced for DA tissue content and enhanced for 5-HT and noradrenaline tissue contents [29]. Similarly, the reduction of correlations was small after WAY-163909 when comparing 5-HIAA/5-HT versus DOPAC/DA, but the profile was very different. At 3 mg/kg, the frontal DOPAC/DA ratios established several correlations with cortical and subcortical 5-HIAA/5-HT ratios. The distinct pattern of effects elicited by 0.3 and 3 mg/kg could have mainly two origins. As far as the 5-HT_2C_ receptors are concerned, it has to be considered that the 5-HT_2C_ receptor mRNA is post-transcriptionally edited at five distinct sites of its coding sequence [57]. Higher levels of editing reduce the affinity for ligands and the coupling efficiency for intracellular signaling pathways [58,59,60]. The profile of editing shows some regional specificity [61], as it could participate in the seemingly biphasic response induced by 0.3 and 3 mg/kg WAY-163909. The second origin for the reported differences could be an action of WAY-163909 on other targets. WAY-163909 has a 20-fold and 46-fold binding selectivity over the 5-HT_2A_ and 5-HT_2B_ receptors, respectively [51]. A progressive involvement of 5-HT_2A_ receptors for which it would behave as an antagonist [51] cannot be excluded.

The correlative approach of metabolism content between regions merits caution. The lack of a reproducible profile in control animals could be due to several factors, including the inter-individual differences which impact the DOPAC/DA and 5-HIAA/5-HT ratios in some brain regions [62], the precise location of the sampled brain regions from an experiment to another, the affective status of the animals, the systematic pooling of both sides of the brain for one brain region, and perhaps the number of animals in one group. In fact, we could not reproduce the correlations of the DOPAC/DA ratios between the striatal territories we previously reported on a large cohort of Wistar rats [39] or in adolescent male C57Bl6 mice [63]. In an almost similar experimental condition using male Sprague Dawley rats, we actually found some correlations of DOPAC/DA between striatal quadrants (including the shell and the core) and fewer negative correlations compared to the present study (unpublished observation). Although we do not have an explanation for these differences, the slightly higher number of animals (10 instead of 8 in the present study) could be an additional explanation as previously discussed [39].

It has been proposed that the 5-HT_2C_ receptor plays the role of neural rheostat shaping networks that overlap in behavioral dimensions, a hypothesis possibly explaining vulnerability to drug abuse [2]. Without strong quantitative effects of WAY-163909 on various electrophysiological, biochemical, or neuroanatomical markers except for the ones we report here, it is tempting to speculate that some changes of correlations induced by WAY-163909 could correspond to a remodeling of neurobiological networks, redefining overlapping networks as receptor fields. WAY-163909 lowered the sensitivity threshold of the basal ganglia to the stimulation of the cingulate cortex [24]. The magnitude of an existing response was not modified by WAY-163909 in contrast to the 5-HT_2B/2C_ receptor agonist Ro 60-0175 [64], and the effect of WAY-163909 on the threshold sensitivity was attributed to an action on the size of segregated circuits of the basal ganglia. This hypothesis deserves attention but would require adequate neuronal/tissue markers. The correlative analysis of monoamine tissue indexes is interesting because it highlights tissue actions which parallel behavioral effects of 5-HT_2C_ receptor agonists on the impulsive/compulsive dimension and cognitive flexibility [2,16,65,66,67], anxious states [68,69,70,71], or food intake [5,52,72,73]. Nevertheless, even if this approach has several dimensions with the study of the neurotransmitter (intraneuronal pool), metabolites, and turnovers, it is only descriptive and barely highlights the mechanisms of interaction of 5-HT_2C_ agonists in the brain.

In conclusion, the 5-HT_2C_ receptor agonist WAY-163909 modified the metabolites and the turnover indexes of 5-HT and DA only in a few brain regions. With an almost similar number of correlations of DOPAC/DA or 5-HIAA/5-HT between pairs of brain regions, WAY-163909 changed the whole pattern of correlations for both indexes. It is possible that the therapeutic actions of 5-HT_2C_ receptor agonists in diseases like obesity, abuse of drugs, or schizophrenia involves multiple changes over neurobiological networks rather than specific effects in one of them [1].

## 4. Materials and Methods

### 4.1. Animals

Male Sprague Dawley rats weighing 300–400 g were used (24 rats in total; Centre d’élevage R Janvier, Le Genest-Saint-Isle, France). They were kept in an animal facility (University of Bordeaux, France) with free access to food and water, in constant temperature (21 ± 2 °C) and humidity (60%) levels, under a 12-h day/night cycle. All the animals’ procedures were in accordance with the European Council Directive 2010/63/EU and the French National Committee (décret 2001-464) and local committee for the care and use of laboratory animals (N°50120130-A, 05/02/2013). All efforts were made to minimize animal suffering and to reduce the number of animals used.

### 4.2. Tissue Sampling and Conditioning

The whole procedure has been recently reported [29]. After the sacrifice, brains were frozen and cut using a cryostat at −24 °C. Bilateral “punches” were made of various brain structures of interest using steel cannulae except for the subthalamic nucleus (STN), which was gently and progressively collected from the surface.

The selected brain regions belonged to different neurobiological networks and were selected with the help of a brain atlas [74]. Selected bilateral pieces of tissue for one brain region were placed in previously weighed Eppendorf tubes and brought to the freezer at −80 °C until the day of the biochemical analysis. Photos were taken throughout to ensure that the samples were taken from a similar plane each time. After the weighing of the filled tube to determine the weight of the tissue, 100 μL of perchloric acid (HClO_4_ 0.1N, 4 °C) was added. The sonication of the samples preceded their centrifugation (13,000 rpm, 30 min, 4 °C), and part of the supernatant was injected into the high-pressure liquid chromatography (HPLC) system.

### 4.3. HPLC Analysis and Electrochemical Detection

The tissue concentrations of monoamines and metabolites were measured by HPLC coupled to the coulometric detection system using conditions previously reported [29]. The monoamines exited the column at different retention times (approximately DOPAC: 5’30; DA: 8’; 5-HIAA: 10’20”; HVA: 15’; 5-HT: 18’). They passed into the coulometric detection cell (Cell 5011, ESA, Paris, France), where the potential of the two electrodes was fixed via the coulometric detector (CoulochemII, ESA, Paris, France) at +350 mV (oxidation) and −270 mV (reduction), respectively. The coulometric detector was connected to a computer through an interface (Ulyss, Azur system, Toulouse, France).

The calibration curves were adapted to the brain areas investigated due to the heterogeneous distribution of monoamines and metabolites across the brain [32,39]. We also adapted a gains set at the level of the detector using a timeline method. The gains used ranged from 5 nA (for the DOPAC, DA, HVA in the hippocampus) to 1 µA (for DA in the striatum). Standard solutions were used before each series of 10/12 samples to verify the good correspondence of the chromatographic conditions. The composition of the standards varied depending on the brain region investigated. The overall sensitivity for the compounds (in pg/10 µL and its expression in fmol): DA, 2.3 (14.5 fmol); DOPAC, 3.4 (20.2); 5-HIAA, 5.5 (28.8); HVA, 18 (98.8); 5-HT, 7.6 (43.1) with a signal/noise ratio of 3:1.

We used two or even three HPLC systems in parallel, which could slightly differ in their sensitivity. In any case, all the samples for one brain region were analyzed with the same HPLC system.

### 4.4. Pharmacological Treatment and Experimental Design

WAY-163909 (7bR,10aR)-1,2, 3,4,8,9,10,10a-octahydro-7bH-cyclopenta-[b][1,4]diazepino[6,7, 1hi]indole]), a generous gift from Dr. John Dunlop (Wyeth Research, USA), was freshly diluted as free base in NaCl 0.9% the day of the experiment. It was injected i.p. (0.3 or 3 mg/kg). For each experimental group, the animals were randomized and received either the drug (0.3 or 3 mg/kg) or appropriate vehicle (in 1 ml/kg). The three treatments were equally distributed for the 24 rats (*n* = 8 for each treatment). The doses of 0.3 and 3 mg/kg, i.p. were chosen based on previous data showing that they reduced locomotor activity and induced purposeless oral movements [48,75]. These studies reported maximal behavioral effects after 30 minutes, and we correspondingly stopped the experiment 45 minutes after its injection at a time corresponding to the highest behavioral activity. We also briefly evaluated abnormal oral movements and penile grooming without scoring them in this experiment.

### 4.5. Statistical Analysis of the Data

The tissue levels of 5-HT and DA for each structure have been reported in a previous article [29]. Here, we report the values expressed in pg/mg for 5-HIAA, DOPAC, and HVA in each brain region. These levels are presented as the mean ± the standard error of the mean (SEM) according to their treatment group. Outlier data were discarded on the basis of the value outside the range of the average mean ± two standard deviations [39], though there were few detected outliers. On the other hand, data were lost due to accidental manipulations (e.g., piece of tissue lost during the punching procedure, aberrant determination of the weight of the piece of tissue). Additionally, several data were lost for HVA due to the poor sensitivity and/or low levels, and groups having lost half of the data (4 observations on 8) were discarded. We calculated the index of the 5-HT and DA turnovers as 5-HIAA/5-HT and DOPAC/DA for each animal and structure and reported the values as mean ± SEM. The values for 5-HIAA, DOPAC, HVA, 5-HIAA/5-HT, and DOPAC/DA were compared between experimental groups (saline, WAY-163909 at 0.3 or 3 mg/kg, i.p.) with a one-way ANOVA, followed by the Fisher protected least significant difference (PLSD) post-hoc test. *p* < 0.05 was used as the criterion for significance.

The qualitative analysis corresponds to multiple correlative analyses using Bravais–Pearson’s correlation coefficient. These analyzes were performed within and between 5-HIAA/5-HT and DOPAC/DA in the 29 brain regions investigated. The correlations were separately performed in rats receiving saline, 0.3 mg/kg WAY-163909, and 3 mg/kg WAY-163909 to estimate connectivity. As previously reported [32], *p*-values were adjusted using the false discovering rate (FDR) controlling procedures. Correlations were then considered as significant at the 5% level and were reported in the corresponding figures. Statistics were done using SigmaPlot 11.0 software.

## Figures and Tables

**Table 1:** Tissue content of 5-HIAA, DOPAC, and homovanillic acid (HVA) in brain regions of saline and WAY-163909-treated rats.

Brain Regions	5-HIAA			DOPAC			HVA		
	Saline	WAY 0.3	WAY 3	Saline	WAY 0.3	WAY 3	Saline	WAY 0.3	WAY 3
**MO**	206 ± 30	224 ± 20	252 ± 19	122 ± 22	121 ± 15	180 ± 22	23.9 ± 3.12	91.4 ± 20 *	103 ± 24 **
**LO**	212 ± 34	188 ± 26	328 ± 54	35.8 ± 2.81	44.1 ± 15.3	66.1 ± 9.22	nd	nd	nd
**DLO**	73.6 ± 11.9	37.1 ± 2.95 *	59.1 ± 11.3	13.2 ± 2.88	12.6 ± 1.8	20.3 ± 3.92	nd	nd	nd
**M2**	92.2 ± 20.1	87.3 ± 12.6	104 ± 15.6	27.6 ± 3.09	20.9 ± 1.7	30.4 ± 5.76	1.82 ± 0.61	1.28 ± 0.34	1.99 ± 0.35
**PL**	137 ± 10.9	149 ± 24.7	116 ± 13.9	9.12 ± 1.30	12.5 ± 3.43	9.71 ± 1.95	36.2 ± 11.7	103 ± 20.3	62.9 ± 19.7
**IL**	147 ± 35.3	124 ± 19.1	121 ± 31.2	29.9 ± 5.16	19.4 ± 1.94	25.5 ± 6.39	18.5 ± 3.92	16.3 ± 3.21	24.6 ± 4.94
**aCg**	336 ± 16.0	312 ± 72.6	228 ± 45.1	180 ± 14.1	118 ± 34.6	115 ± 27.5	nd	nd	nd
**pCg**	386 ± 43.8	348 ± 34.5	308 ± 16.8	41.9 ± 3.28	38.0 ± 4.49	30.3 ± 1.46	nd	nd	nd
**ains**	136 ± 18.9	113 ± 20	106 ± 11.8	24.5 ± 5.13	27.9 ± 4.72	21.2 ± 2.4	8.86 ± 2.75	6.67 ± 1.22	6.15 ± 0.71
**pins**	99.6 ± 8.35	79 ± 11.7	102 ± 15.9	17.1 ± 4.1	15.2 ± 1.6	16.9 ± 3.63	3.98 ± 0.56	3.75 ± 0.76	4.22 ± 0.70
**dHP**	81.8 ± 9.15	60.3 ± 8.36	81.8 ± 4.75	12.3 ± 0.34	14.24 ± 0.90	12.43 ± 0.67	nd	nd	nd
**vHP**	159 ± 3.61	139 ± 17.6	132 ± 11.9	15.2 ± 2.2	15.5 ± 3.04	13.71 ± 4.2	nd	nd	nd
**BLA**	151 ± 17.7	215 ± 25.9	229 ± 17.5	104 ± 53.9	116 ± 23.6	100 ± 32.7	nd	nd	nd
**CE**	132 ± 18.6	199 ± 21.4 *	108 ± 19.9	59.0 ± 10.0	131 ± 40.3	45.8 ± 13.9	nd	nd	nd
**shell**	178 ± 17.5	123 ± 10.5	154 ± 16.1	728 ± 85.8	595 ± 73.0	726 ± 71.0	540 ± 83.6	449 ± 41.4	586 ± 52.8
**core**	76.0 ± 6.36	63.8 ± 7.52	73.0 ± 11.1	847 ± 78	682 ± 106	825 ± 145	150 ± 9.02	124 ± 16.1	166 ± 23.5
**VMS**	142 ± 23.8	137 ± 13.8	170 ± 23.5	480 ± 80.2	477 ± 72.6	569 ± 84.6	257 ± 56.7	272 ± 48.4	352 ± 107
**VLS**	208 ± 12.3	244 ± 18.2	274 ± 28.2	876 ± 123	661 ± 49.6	799 ± 58.6	407 ± 62.8	363 ± 15.2	445 ± 36.6
**DMS**	96.2 ± 15.9	85.6 ± 6.45	79.1 ± 7.23	709 ± 109	709 ± 98.6	720 ± 83.1	603 ± 95.8	667 ± 66.4	806 ± 59.0
**DLS**	190 ± 66.9	110 ± 17.6	144 ± 20.3	797 ± 129	727 ± 92.5	838 ± 110	982 ± 93.3	827 ± 97.0	1113 ± 167
**VCS**	172 ± 36.9	173 ± 38.4	250 ± 42.2	307 ± 65.4	272 ± 76.9	471 ± 82.6	359 ± 80.6	359 ± 96.8	521 ± 94.2
**EPN**	147 ± 17.2	174 ± 25.8	111 ± 15.4	16.7 ± 2.92	18.6 ± 3.81	11.40 ± 2.11	1.76 ± 0.27	2.38 ± 0.65	1.79 ± 0.23
**STN**	257 ± 43.4	260 ± 37.6	310 ± 34.9	132 ± 32.5	148 ± 22.6	181 ± 29.7	6.48 ± 2.15	7.57 ± 1.29	9.05 ± 1.40
**vHY**	74.9 ± 8.82	54.3 ± 6.15	51.8 ± 8.67	28.2 ± 7.14	22.8 ± 4.02	25.0 ± 3.40	nd	nd	nd
**dHY**	156 ± 23.6	101 ± 14.9	81 ± 21.6	3.16 ± 0.62	2.19 ± 0.52	2.41 ± 0.63	2.63 ± 0.77	1.86 ± 0.57	3.87 ± 1.29
**SN**	738 ± 55.9	766 ± 47.9	754 ± 37.8	474 ± 82.4	436 ± 70.1	642 ± 61.7	194 ± 59.8	133 ± 34.5	176 ± 50.9
**VTA**	428 ± 44.3	459 ± 41.4	385 ± 62.8	625 ± 64.3	650 ± 91.4	597 ± 67.6	83.0 ± 10.8	85.0 ± 8.17	75.4 ± 10.5
**DR**	396 ± 32.6	373 ± 19.8	407 ± 33.1	23.2 ± 5.9	19.2 ± 3.9	31.9 ± 7.3	nd	nd	nd
**MR**	318 ± 62	230 ± 35	265 ± 40.5	63.9 ± 18.8	107 ± 28.8	37.9 ± 19.2	15.4 ± 1.8	16.4 ± 2.9	9.13 ± 1.7

Data for 5-HIAA, DOPAC, and HVA are mean ± SEM values (pg/mg of tissue) per group in the 29 brain regions. The number of values retained in each group in each group has been reported in Table S1. It usually comprises between 6–8 values/group with some exceptions (notably: DOPAC in LO for saline-treated rats, n = 4; DOPAC in dHP for 3 mg/kg, *n* = 3). * *p* < 0.05, ** *p* < 0.01 (PLSD test) compared to saline-treated rats. nd—not detected.

## Supplementary Materials

The following are available online at https://www.mdpi.com/1422-0067/20/12/2925/s1.

## Abbreviations

aCg	anterior cingulate cortex
ains	anterior insular cortex
BLA	basolateral nucleus of the amygdala
CE	central nucleus of the amygdala
CNS	central nervous system
core	core of the nucleus accumbens
DA	dopamine
dHP	dorsal hippocampus
DLO	dorsolateral orbitofrontal cortex
DLS	dorsolateral striatum
DMS	dorsomedial striatum
DOPAC	3,4-dihydroxyphenylacetic acid
DR	dorsal raphe nucleus
dHY	dorsal hypothalamus
EPN	entodepuncular nucleus
HPLC	high pressure liquid chromatography
IL	infralimbic cortex
LO	lateral orbitofrontal cortex
M2	motor cortex M2
MO	medial orbitofrontal cortex
MR	median raphe nucleus
pCg	posterior cingulate cortex
pins	posterior insular cortex
PL	prelimbic cortex
5-HIAA	5-hydroxyindole-3-acetic acid
5-HT	5-hydroxytryptamine; serotonin
5-HT_2C_ receptors	Serotonin 2C receptors
shell	shell of the nucleus accumbens
SN	substantia nigra
STN	subthalamic nucleus
vHP	ventral hippocampus
vHY	ventral hypothalamus
VCS	ventrocaudal striatum
VLS	ventrolateral striatum
VMS	ventromedial striatum
VTA	ventral tegmental area
WAY-163909	(7bR,10aR)-1,2,3,4,8,9,10,10a-octahydro-7bH-cyclopenta-[b][1,4]diazepino[6,7, 1hi]indole]
