# Supplementary material for: Effect of the 5-HT2C Receptor Agonist WAY-163909 on Serotonin and Dopamine Metabolism across the Rat Brain: A Quantitative and Qualitative Neurochemical Study
Source: Int J Mol Sci. 2019 Jun 14;20(12):2925. doi: 10.3390/ijms20122925 (PMC6627111; doi:10.3390/ijms20122925)
Supplement: Supplementary file 1 [file ijms-20-02925-s001.pdf]

Effect of the 5-HT_{2C} Receptor Agonist WAY-163909 on Serotonin and Dopamine Metabolism across the Rat Brain: A Quantitative and Qualitative Neurochemical Study

Sara Whitestone[1*], Philippe De Deurwaerdère[1*], Lynn Baassiri[1], Julien Manem[1], Youssef Anouar[2], Giuseppe Di Giovanni[3,4], Rahul Bharatiya[1,5], Abdeslam Chagraoui[2*]

SUPPLEMENTARY FILE

Table S1: Experimental details.

Brain regions	Abbreviation	values (n)/group (Saline, WAY-163909 at 0.3 and 3 mg/kg)				
		5-HIAA	DOPAC	HVA	5-HIAA/5-HT	DOPAC/DA
Orbital Cortex	OFC					
medial orbital	MO	8,8,8	8,8,8	8,8,8	8,8,8	8,8,7
Lateral orbital	LO	8,7,8	4*,7,6	nd	7,6,8	4*,7,6
Dorsolateral orbital	DLO	8,8,8	5,6,6	nd	8,8,8	5,6,6
Motor M2	M2	8,7,7	7,7,7	7,6,7	8,7,7	7,7,7
Frontal Cortex						
prelimbic	PL	8,8,8	8,8,8	4,5,6	8,8,8	8,8,8
infralimbic	IL	8,8,8	8,7,8	8,8,8	8,8,8	8,7,8
Anterior cingulate	aCg	8,8,8	7,7,7	nd	8,7,8	7,7,7
Posterior cingulate	pCg	8,8,8	8,8,8	nd	8,8,8	8,8,8
Anterior insular	ains	6,7,6	6,7,6	6,7,6	6,7,6	6,7,6
Posterior insular	pins	6,8,8	6,8,8	6,8,8	6,8,8	6,8,7
Nucleus accumbens Striatum	NAc					
Shell	Shell	8,8,8	8,8,8	8,8,8	8,8,8	8,8,8
core	core	8,7,8	8,7,8	8,7,8	8,7,8	8,7,8
Dorsomedial striatum	DMS	8,8,8	8,8,8	8,8,8	8,8,8	8,8,8
Ventromedial striatum	VMS	8,8,8	8,8,8	8,8,8	8,8,8	8,8,8
Dorsolateral striatum	DLS	8,8,8	8,8,8	8,8,8	8,8,8	8,8,8
Ventrolateral striatum	VLS	8,8,8	8,8,8	8,8,8	8,8,8	8,8,8
Ventrocaudal striatum	VCS	7,8,8	8,8,8	8,8,8	7,8,8	8,8,8
Basal ganglia mesencephalon						
Entopuncular nucleus	EPN	8,7,8	8,8,8	8,8,8	8,7,8	8,8,8
Subthalamic nucleus	STN	8,8,8	8,8,8	6,6,7	8,8,8	8,8,8
Substantia nigra	SN	8,8,8	8,8,8	8,8,8	8,8,8	8,8,8
Ventral tegmental area	VTA	8,8,8	8,8,8	8,8,8	8,8,8	8,8,8
Dorsal raphe nucleus	DR	7,8,8	7,7,8	nd	7,8,8	7,7,8
Median raphe nucleus	MR	8,8,8	7,8,6	7,6,6	7,7,6	7,7,6
Amygdala						
Basolateral nucleus	BLA	8,8,8	8,8,8	nd	8,8,8	8,8,8
Central nucleus	CE	8,7,8	8,8,8	nd	8,7,7	8,8,8
Hippocampus						
Dorsal, anterior parts	dHP	8,8,8	7,5,3*	nd	8,8,8	7,5,3*
Ventral, posterior parts	vHP	6,8,7	6,6,5	nd	6,8,7	5,6,5
Hypothalamus						
dorsal parts	dHY	7,8,8	7,6,8	5,6,6	7,8,8	7,6,8
Ventral parts	vHY	8,8,8	7,8,7	nd	8,8,8	7,8,7

The 3 numbers reported in the columns (dependent variable 5-HIAA, DOPAC, HVA, 5-HIAA/5-HT, and DOPAC/DA) correspond to the number of values considered for the statistical analysis for each brain regions. Starting from 8 observations/group, lower numbers are due to loss of tissue, accidental manipulations, loss of chromatographic signal and/or outliers. The asterisk* highlights two groups of data that were not considered for correlations due to the low number of data. nd - not detected.
